# Supplementary material for: Content-rich biological network constructed by mining PubMed abstracts
Source: BMC Bioinformatics. 2004 Oct 8;5:147. doi: 10.1186/1471-2105-5-147 (PMC528731; doi:10.1186/1471-2105-5-147)
Supplement: Additional File 5 — The original Chilibot query results of the term "long-term potentiation (LTP)" and 22 other terms, limiting the latest references analyzed to the years 1990, 1995, 2000, and 2004. [file 1471-2105-5-147-S5.bz2 › chilibotAdditionalFile5/ltp2000/html/TRKA_ARC.html]

 


 **TRKA** and **ARC** 
  
Found 2 abstracts in PubMed,  **2 abstracts were retrieved and analyzed**.  


---

 Search Google  |
 PDF files only 
|  EDU domain only 

---

**Interactive relationship** (e.g. stimulation, inhibition, etc)

- The arcA gene product or the  **Arc**  system is proposed to be implicated in the regulation of the relationship between  **TrkA**  and F0F1.  Ref: 9798786 Biosci Rep, 1998
- The  **Arc**  system for the control of synthesis of respiratory enzymes in E. coli participates in the regulation of the K transporting  **TrkA**  system, which directly interacts with F0F1 H ATPasa.  Ref: 9702339 BiofizikaBiofizika, 1998
